# The value of diastolic function parameters in the prediction of left atrial appendage thrombus in patients with nonvalvular atrial fibrillation

**DOI:** 10.1186/1476-7120-12-10

**Published:** 2014-02-25

**Authors:** Rami Doukky, Enrique Garcia-Sayan, Heather Gage, Vijaiganesh Nagarajan, Anna Demopoulos, Marek Cena, Noreen T Nazir, George J Karam, Richard G Trohman, Rasa Kazlauskaite

**Affiliations:** 1 Division of Cardiology, Rush University Medical Center, 1653 W. Congress Pkwy, Chicago, IL 60612, USA; 2 Division of Adult Cardiology, John H. Stroger, Jr. Hospital of Cook County, Chicago, IL, USA; 3 Division of Cardiology, Mount Sinai Hospital, Chicago, IL, USA; 4 Department of Preventive Medicine, Rush University Medical Center, Chicago, IL, USA

**Keywords:** Diastolic function, Left atrial appendage thrombus, Spontaneous echo contrast (SEC), Atrial fibrillation

## Abstract

**Background:**

Left ventricular diastolic impairment and consequently elevated filling pressure may contribute to stasis leading to left atrial appendage thrombus (LAAT) in nonvalvular atrial fibrillation (AF). We investigated whether transthoracic echocardiographic parameters can predict LAAT independent of traditional clinical predictors.

**Methods:**

We conducted a retrospective cohort study of 297 consecutive nonvalvular AF patients who underwent transthoracic echocardiogram followed by a transesophageal echocardiogram within one year. Multivariate logistic regression analysis models were used to determine factors independently associated with LAAT.

**Results:**

Nineteen subjects (6.4%) were demonstrated to have LAAT by transesophageal echocardiography. These patients had higher mean CHADS_2_ scores [2.6 ± 1.2 vs. 1.9 ± 1.3, *P* = 0.009], higher E:e’ ratios [16.6 ± 6.1 vs. 12.0 ± 5.4, *P* = 0.001], and lower mean e’ velocities [6.5 ± 2.1 cm/sec vs. 9.1 ± 3.2 cm/sec, *P* = 0.001]. Both E:e’ and e’ velocity were associated with LAAT formation independent of the CHADS_2_ score, warfarin therapy, left ventricular ejection fraction (LVEF), and left atrial volume index (LAVI) [E:e’ odds-ratio = 1.14 (95% confidence interval = 1.03 – 1.3), *P* = 0.009; e’ velocity odds-ratio = 0.68 (95% confidence interval = 0.5 – 0.9), *P* = 0.007]. Similarly, diastolic function parameters were independently associated with spontaneous echo contrast.

**Conclusion:**

The diastolic function indices E:e’ and e’ velocity are independently associated with LAAT in nonvalvular AF patients and may help identify patients at risk for LAAT.

## Background

**Figure 1:**
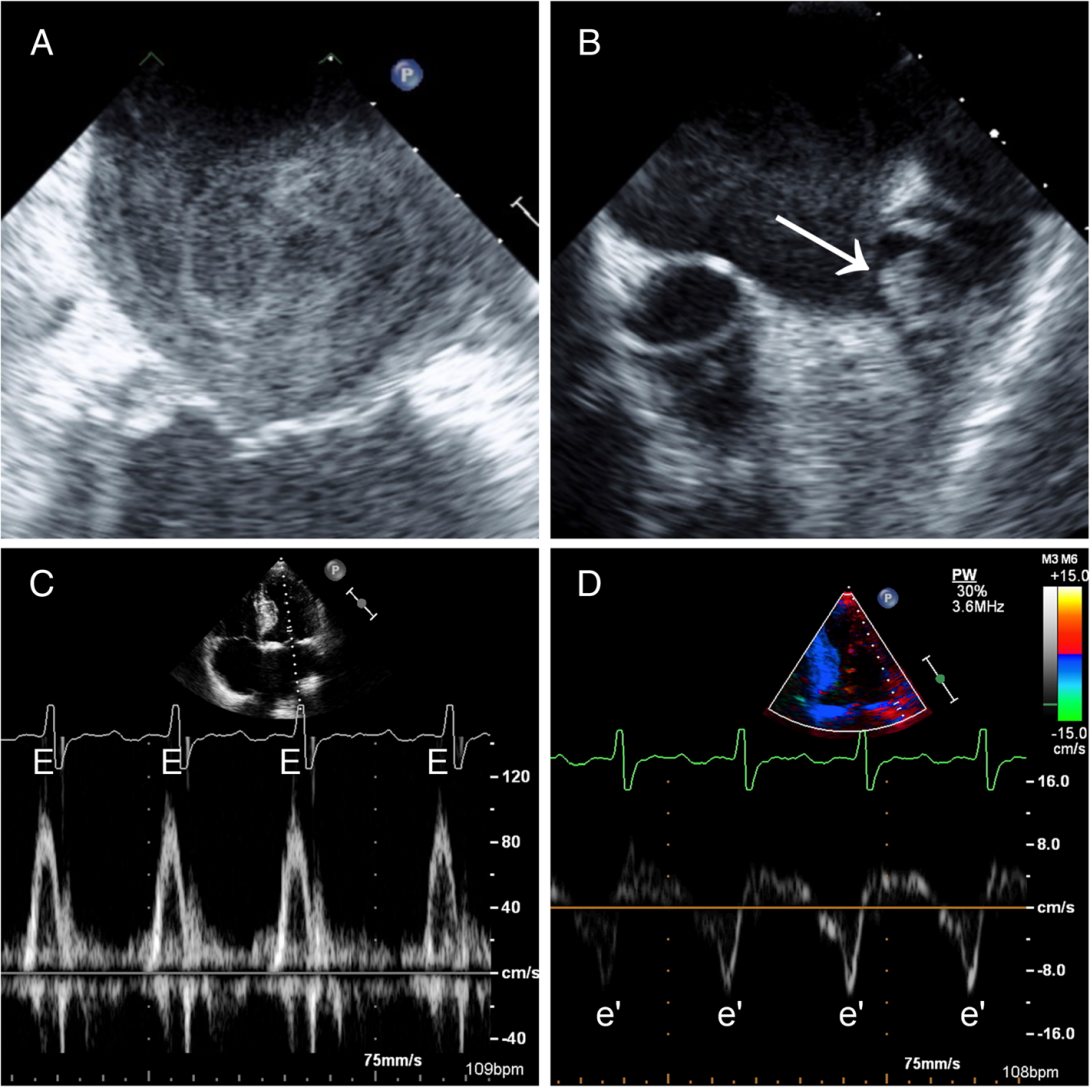
**Representative examples. A**: left atrial spontaneous echo contrast (SEC). **B**: left atrial appendage thrombus (arrow). **C**: Pulsed-wave Doppler recording of early diastolic mitral inflow velocity (E). **D**: Spectral tissue Doppler imaging of the lateral mitral annulus early diastolic velocity (e’).

Nonvalvular atrial fibrillation (AF) is the most common sustained cardiac dysrhythmia and the most frequent cause of cardio-embolic stroke [1]. It is well known that left atrial appendage thrombi (LAAT) are the source of most embolic strokes in patients with nonvalvular AF [2,3]. It is, likewise, widely accepted that transesophageal echocardiography (TEE) can identify LAAT and left atrial blood stasis, manifesting as spontaneous echo contrast (SEC), a known precursor of LAAT and systemic thromboembolism (Figure 1) [4-6].

It is physiologically plausible that impaired diastolic function and consequent elevation in the left ventricular filling pressure (LVFP) lead to left atrial stasis which results in LAAT formation and subsequent risk of systemic thromboembolism. Prior reports suggest that two-dimensional echocardiographic parameters such as left ventricular ejection fraction (LVEF), left atrial volume index (LAVI) and LVEF/LAVI are associated with LAAT in patients with AF [7,8]. In this investigation, we sought to determine whether e’ velocity (as a surrogate for left ventricular relaxation) and E:e’ (as a measure of left ventricular filling pressure) are predictive of LAAT formation and SEC independent of confounding covariates. Furthermore, we sought to integrate diastolic function indices with other echocardiographic parameters to propose a prediction rule for LAAT in patients with nonvalvular AF.

## Methods

### Patient population and study design

A retrospective cohort study design was implemented. We queried the echocardiography laboratory database at Rush University Medical Center to identify all consecutive adult patients with nonvalvular AF who underwent a TEE to “rule-out” left atrial appendage thrombus between January 1, 2005 and December 31, 2009. Of those, we only included patients who had a previous transthoracic echocardiogram (TTE) within 1 year of the TEE. Patients with atrial flutter, without intervening episodes of atrial fibrillation, were not included. We excluded patients with valvular AF due to mitral stenosis and those with conditions known to alter E and e’ velocities; namely mitral regurgitation greater than 2+ in severity (on a 0 to 4 scale), post mitral valve surgical or percutaneous intervention, and post orthotopic heart transplantation status. Patients with isolated aortic valvular disease, aortic valve prostheses and right-sided valvular heart disease were not excluded [1].

An expert board-certified (NBE) echocardiographer (RD), who was blinded to the TTE and clinical data, reviewed all TEE images to determine the presence or absence of LAAT [LAAT(+) and LAAT(−)], SEC and depressed left atrial appendage emptying velocity (<40 cm/sec) by pulsed-wave Doppler. LAAT was defined as a circumscribed and uniformly echodense intracavitary mass distinct from the underlying left atrial or left atrial appendage endocardium and the pectinate muscles, and present in more than one imaging plane [9]. Left atrial appendage sludge, defined as a dynamic gelatinous, precipitous echodensity, without a discrete mass, present throughout the cardiac cycle was categorized as LAAT [10,11]. SEC was defined as dynamic “smoke-like” echoes with the characteristic swirling motion with optimal gain setting during the entire cardiac cycle [12].

All TTEs were reviewed offline by a board-certified (NBE) echocardiographer who was blinded to TEE and clinical data (HG). The mitral inflow early-diastolic pulsed-wave Doppler velocity (E) and the lateral mitral annulus tissue Doppler early-diastolic velocity (e’) were measured (cm/sec) and the E:e’ ratio was calculated (Figure 1). We did not analyze e’ velocities sampled from the medial mitral annulus, since this was not routinely obtained according to our laboratory protocol at the time when the study TTEs were performed (2005–2009). The Doppler measurements were obtained by averaging data from 3–5 consecutive beats. The left ventricular septal and posterior wall thicknesses and end-diastolic as well as end-systolic internal dimensions were measured from 2-dimentional and M-mode TTE images [13]. The left ventricular mass was calculated using the Devereux formula [14]. The left ventricular systolic and diastolic volumes and LVEF were measured using the biplane Simpson’s method or the Teichholz formula only when the former was not feasible due to suboptimal apical views [13,15]. The left atrial dimensions (antero-posterior, medio-lateral and supero-inferior) were measured and the left atrial volume was calculated using the formula [4/3π (D1/2) (D2/2) (D3/2)] where each “D” represents one of three atrial dimensions [13]. All volume and mass measurements were indexed to the body surface area. The heart rhythm during TTE acquisition was determined from examination of the rhythm strip on the screen display.

Finally, a detailed chart review was performed to collect data on demographics, comorbidities, anticoagulant and antiplatelet use, and INR (international normalized ratio). Congestive heart failure (CHF) was defined as any clinical heart failure or LVEF < 40%. Date-of-onset and chronicity of AF were determined from patients’ health records. Paroxysmal AF was defined as AF spontaneously reverting to sinus rhythm within 7 days from onset. Persistent AF was defined as AF lasting more than 7 days, but less than 6 months, or any AF events terminated by electrical or chemical cardioversion or radiofrequency ablation within 6 months from onset. Permanent AF was defined as lasting more than 6 months [1]. The CHADS_2_ score was calculated from the sum of risk predictors of CHF, hypertension, age ≥75, diabetes mellitus, stroke or transient ischemic attack (TIA); weighing each by “1” except for prior stroke/transient ischemic attack (TIA) which was weighed by “2” [16]. The CHA_2_DS_2_-VASc score was calculated from the sum of the risk factors of CHF, hypertension, age 65–74 or ≥75 years, diabetes mellitus, stroke/TIA, vascular disease, female gender; weighing each by “1” except for stroke/TIA and age ≥75 which were weighed by “2” [17]. The primary outcome of the study was TEE-identified LAAT. The secondary outcome was TEE-identified SEC.

### Statistical analysis

The two-tailed Student’s *t*-test was used to compare normally-distributed continuous variables which were expressed as mean ± standard deviation (SD). The Mann–Whitney-U test was used to compare continuous variables that did not adhere to a normal distribution which were expressed as median (interquartile range). The Shapiro-Wilk test was used to confirm the normality of data. The chi-square (χ^2^) test was used to compare categorical variables which were expressed as frequency [n (%)]. The Spearman’s method was used to evaluate linear correlations.

Multivariate logistic regression analysis models were used to determine clinical and echocardiographic predictors of outcome measures independent of known confounders. Risk was expressed as odd-ratios (OR) with 95% confidence intervals (CI). The Hosmer-Lemeshow test was used to test regression models’ goodness of fit.

The receiver operating characteristic (ROC) methodology was used to analyze the discriminatory capacity of various predictors of LAAT. ROC analyses were expressed as curve plots with the associated area under the curve (AUC) with 95% CI and a *P* value representing the likelihood of the null hypothesis (AUC = 0.5). A two-tailed *P* value ≤0.05 was considered statistically significant in all analyses. PASW-18 software (SPSS, Inc. - Chicago, IL) was used for all data analyses with the exception of the comparisons between ROC curves for which STATA-11 (College Station, TX) was used. The study was funded by an internal research grant and was approved by the Rush University Medical Center institutional review board.

## Results

A database query yielded 527 TEEs performed in patients with nonvalvular AF to “rule-out” LAAT. Of those, 167 were excluded due to mitral valvular disease and 63 for not having had a TTE performed at our institution within a year prior to the TEE. Consequently, a cohort of 297 consecutive TEEs in adult subjects with nonvalvular AF met the inclusion criteria. The electrophysiological procedures that prompted the TEE were: cardioversion in 131 (44.1%), AF ablation in 99 (33.3%), implantation and testing of an implantable cardioverter-defibrillator in 45 (15.2%), and revision and testing of an implantable cardioverter-defibrillator in 22 (7.4%) subjects. The median interval between the TTE and TEE was 7.5 days (interquartile range 2–93 days). The mean INR within 30 days prior to the TEE for warfarin-treated patients was 1.7 ± 0.8. Nineteen subjects (6.4%) had a TEE proven LAAT. At the time of the TTE, 98 (33%) subjects were in sinus rhythm, 178 (60%) in AF or atrial flutter, and 21 (7%) in paced ventricular rhythm. Notably, patients with LAAT had a significantly higher mean CHADS_2_ score, along with an increase in warfarin use (Table 1). Prior to the TEE, adequate anticoagulation was confirmed in 14 (74%) subjects with LAAT, and twelve of these (86%) had a CHADS_2_ score ≥2. AF chronicity and duration were not different between those with and without LAAT (Table 1).

**Table 1:** Baseline clinical characteristics

	**LAAT (+)**	**LAAT (−)**	***P *** **value**
	**N = 19**	**N = 278**	
**Age (years), mean ± SD**	66 ± 13	62 ± 14	0.37
**Age ≥ 75 years, n (%)**	5 (26%)	55 (20%)	0.49
**Male gender, n (%)**	14 (74%)	176 (63%)	0.36
**Hypertension, n (%)**	16 (84%)	177 (64%)	0.07
**Diabetes mellitus, n (%)**	8 (42%)	73 (26%)	0.13
**Congestive heart failure, n (%)**	15 (79%)	132 (47%)	0.01
**History of stroke or TIA, n (%)**	2 (11%)	27 (10%)	0.91
**Aspirin, n (%)**	9 (47%)	122 (44%)	0.79
**Clopidogrel, n (%)**	2 (11%)	16 (6%)	0.41
**Antiplatelet, n (%)**	10 (53%)	126 (46%)	0.56
**Warfarin, n (%)**	15 (79%)	148 (53%)	0.03
**CHADS**_**2 **_**score, mean ± SD**	2.6 ± 1.2	1.9 ± 1.3	0.009*
**CHA**_**2**_**DS**_**2**_**-VASc Score, mean ± SD**	3.5 ± 1.7	2.8 ± 1.8	0.08*
**CHADS**_**2 **_**Score ≥ 2, n (%)**	17 (89%)	158 (57%)	0.005
**Duration since first AF episode (days), median (interquartile range)**	691.5 (90–1163)	162 (4–1249)	0.28*
**AF type, n (%)**			
• Paroxysmal	8 (42%)	153 (55%)	0.27
• Persistent	4 (21%)	58 (21%)	0.98
• Permanent	7 (37%)	67 (24%)	0.21
**Creatinine (mg/dL), mean ± SD**	1.6 ± 0.7	1.4 ± 1.0	0.047*

**LAAT:** left atrial appendage thrombus; **SD:** standard deviation; **TIA:** transient ischemic attack; **AF:** atrial fibrillation. *Mann–Whitney U test.

Compared to the 297 analyzed subjects, the 63 patients excluded for lacking a qualifying TTE had no significant differences in the prevalence of LAAT [3 (4.8%) vs. 19 (6.4%), *P* = 0.62], mean age (59 ± 17 vs. 63 ± 14 years, *P* = 0.13), mean CHADS_2_ score (2.1 ± 1.3 vs. 1.9 ± 1.3, *P* = 0.20), and gender distribution [41 (65%) vs. 190 (64%) men, *P* = 0.87]. Furthermore, they had a similar prevalence of CHF, age ≥75 years, diabetes, stroke/TIA history, and warfarin use (all *P* values >0.18).

### E:e’ and e’ velocity association with LAAT

**Figure 2:**
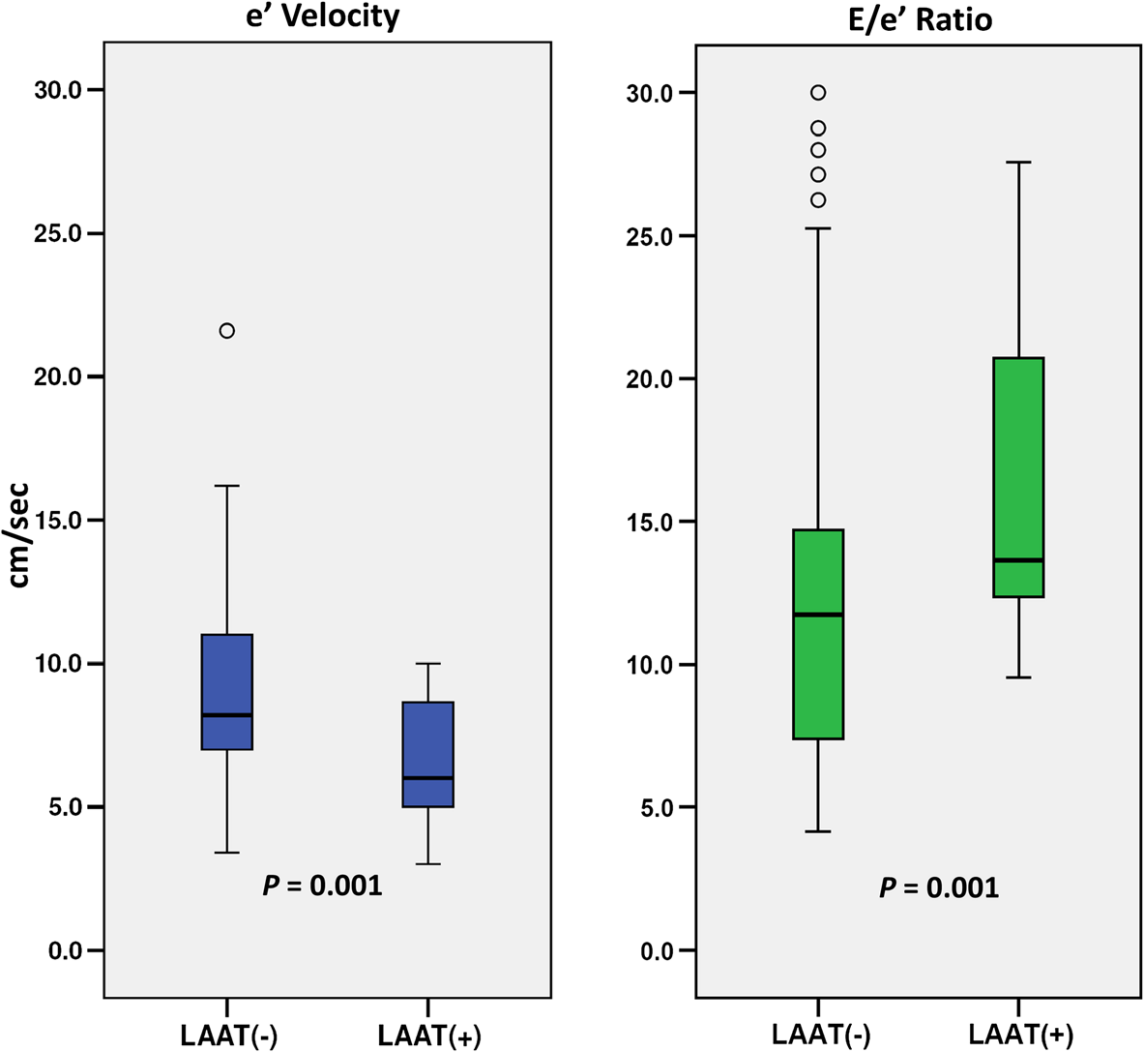
Box plots: E:e’ ratio and e’ velocity.

**Figure 3:**
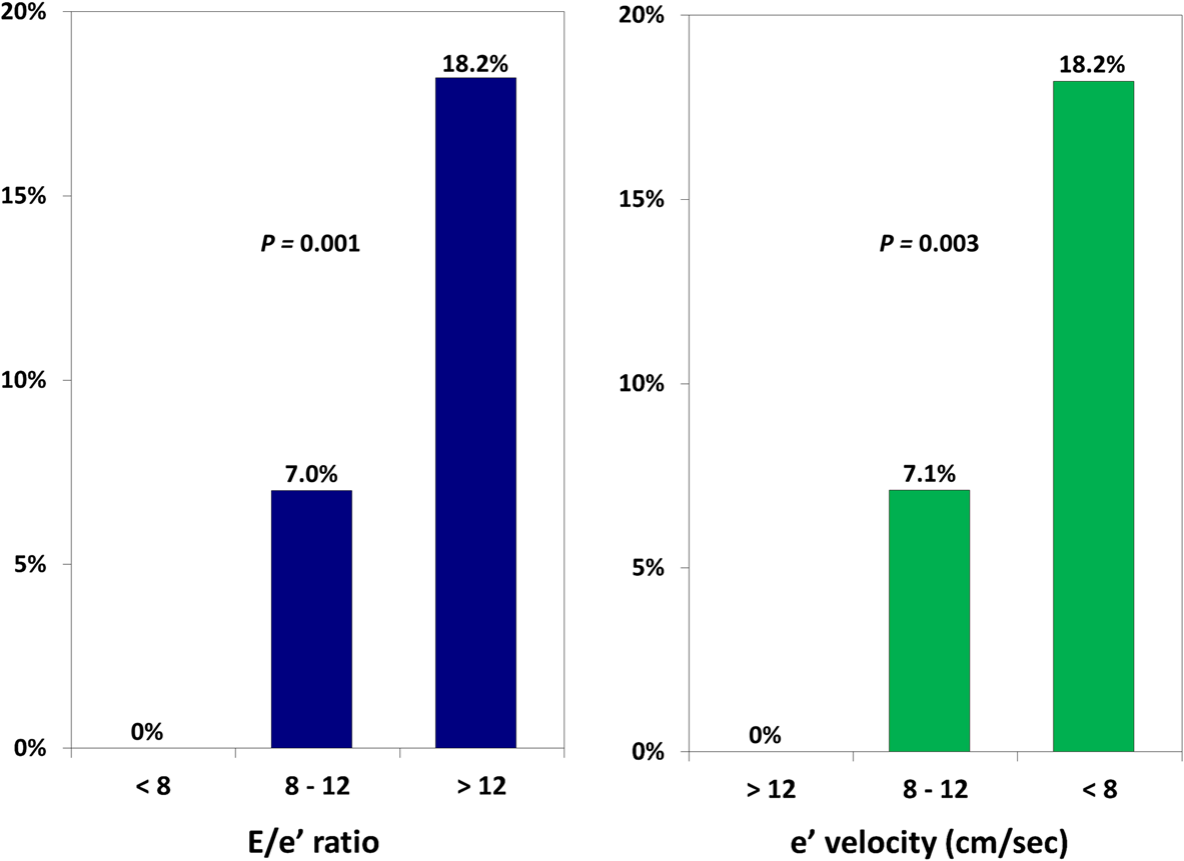
Prevalence of left atrial appendage thrombus based on E:e’ ratio and e’ velocity.

The mean E:e’ among LAAT(+) patients was significantly higher than those who were LAAT(−) [16.6 ± 6.1 vs. 12.0 ± 5.4, respectively; *P* = 0.001]. Conversely, the e’ velocity was significantly lower among LAAT(+) subjects [6.5 ± 2.1 vs. 9.1 ± 3.2, *P* = 0.006] (Figure 2, Table 2). Among LAAT(+) subjects, none (0%) had a normal E:e’ of ≤8; whereas 16 (84%) had an E:e’ ≥ 12, indicative of elevated LVFP [18]. Furthermore, there was a stepwise increase in the prevalence of LAAT with increasing E:e’ and decreasing e’ velocity (Figure 3). Additionally, LAAT(+) subjects had a significantly higher LAVI and left ventricular volume index but lower LVEF (Table 2). Not surprisingly, there was a modest, but highly statistically significant, linear correlation between the CHADS_2_ score and diastolic indices of the E:e’ and e’ velocity, with Spearman’s correlation coefficients “r” of 0.34 (*P* < 0.001) and −0.32 (*P* < 0.001), respectively. Since only a minority of the patients (33%) were in sinus rhythm at the time of TTE, and thus had no A wave in the mitral inflow Doppler waveforms, it was not feasible to classify the patients based on their grade of diastolic dysfunction (typically graded from I to IV).

**Table 2:** Univariate analysis: Echocardiographic parameters

	**LAAT (+)**	**LAAT (−)**	***P *** **value**
	**N = 19**	**N = 278**	
**E:e’ ratio, mean ± SD**	16.6 ± 6.1	12.0 ± 5.4	0.001
**E:e’ > 8, n (%)**	19 (100%)	195 (70%)	0.006
**E:e’ ≥ 12, n (%)**	16 (84%)	127 (46%)	0.001
**e’ velocity (cm/sec), mean ± SD**	6.5 ± 2.1	9.1 ± 3.2	0.001
**Spontaneous echo contrast, n (%)**	19 (100%)	95 (34%)	< 0.001
Depressed LAA emptying velocity*, n (%)	19 (100%)	132 (48.0%)	< 0.001
**LA volume index (mL/m**^**2**^**), mean ± SD**	44 ± 13	30 ± 13	< 0.001
**LV volume index (mL/m**^**2**^**), mean ± SD**	96 ± 38	73 ± 35	0.01
**LV mass index (g/m**^**2**^**), mean ± SD**	124 ± 45	124.0 ± 44	1.0
**LVEF (%), mean ± SD**	26 ± 17	45 ± 20	< 0.001

**LAAT:** left atrial appendage thrombus; **SD:** standard deviation; **LAA:** left atrial appendage; **LA:** left atrial; **LV:** left ventricular; **LVEF:** left ventricular ejection fraction.

*LAA emptying velocity < 40 cm/sec.

A multivariate logistic regression analysis demonstrated that the CHADS_2_ score is associated with LAAT independent of warfarin use [OR = 1.47 per one point CHADS_2_ score increment (CI = 1.04-2.1), *P* = 0.03], while warfarin use had a borderline association with LAAT (Table 3; Model-1). When E:e’ was added to Model-1, it was independently associated with LAAT [OR = 1.14 per 1 point increment (CI = 1.05-1.2), *P* =0.002] and negated the effect of the CHADS_2_ score (Model-2, Table 3). To ensure that this association is not simply a confounder to impaired left ventricular function and left atrial volume, we added LVEF and LAVI to the model forming Model-3, in which E:e’, LVEF and LAVI were independently associated with LAAT, whereas the value of the CHADS_2_ and warfarin use was further negated (Table 3, Model-3).

**Table 3:** Multivariate analysis: Predictors of left atrial appendage thrombus

		**Odds-Ratio**	**95% CI**	***P *** **value**
**Clinical**	**Model 1**			
	CHADS_2_ Score (per 1 point)	1.47	1.04 – 2.1	0.03
	Warfarin	3.1	1.0 – 9.6	0.051
**Clinical, E/e’, and 2-D echo**	**Model-2**			
	E/e’ (per 1 point)	1.14	1.05 – 1.2	0.002
	CHADS_2_ score (per 1 point)	1.36	0.9 – 2.0	0.10
	Warfarin	3.5	1.05 – 11.8	0.04
	Model-3*			
	E/e’ (per 1 point)	1.14	1.03 – 1.3	0.009
	LVEF (per 10 point)	0.62	0.4 – 0.9	0.008
	LAVI (per 10 mL/m^2^)	1.59	1.1 – 2.3	0.02
	CHADS_2_ score (per 1 point)	0.93	0.6 – 1.5	0.74
	Warfarin	2.73	0.7 – 10.7	0.15
**Clinical, e’ velocity, and 2-D echo**	**Model 4**			
	e’ velocity (per 1 cm/sec)	0.70	0.6 – 0.9	0.004
	CHADS_2_ score (per 1 point)	1.27	0.9 – 1.8	0.20
	Warfarin	2.87	0.9 – 9.5	0.08
	Model 5*			
	e’ velocity (per 1 cm/sec)	0.68	0.5 – 0.9	0.007
	LVEF (per 10 point)	0.67	0.5 – 1.01	0.03
	LAVI (per 10 mL/m^2^)	1.89	1.3 – 2.8	0.002
	CHADS_2_ score (per 1 point)	0.86	0.5 – 1.4	0.53
	Warfarin	2.1	0.6 – 7.9	0.26

**CI:** confidence intervals; **LVEF:** left ventricular ejection fraction (%); **LAVI:** left atrial volume index (mL/m^2^). *The Hosmer and Lemeshow test of the final models (3 and 5) showed good fit (*P* values = 0.91and 0.76, respectively).

Similarly, when e’ velocity then LVEF and LAVI were sequentially introduced to Model-1 (Models 4 and 5), e’ velocity was independently “protective” of LAAT after adjusting for other covariates [OR = 0.68 for each 1 cm/sec increment in e’ velocity, *P* = 0.007] while the CHADS_2_ score was not (Table 3; Model-5). The Hosmer and Lemeshow test demonstrated a good fit of Models 3 and 5 (Table 3). Nearly identical results were obtained when analyzing the CHA_2_DS_2_-VASc score instead of CHADS_2_ score.

Additionally, E:e’ and e’ velocity were also associated with LAAT independent of the individual components of CHADS_2_ score [E:e’ odds-ratio = 1.07 per 1 point increment (CI = 1.01-1.14), *P* = 0.02; e’ velocity odds-ratio = 0.87 per 1 cm/sec increment (CI = 0.77-0.98), *P* = 0.03]. These models maintained a good model fit (Hosmer and Lemeshow test *P* values = 0.30 and 0.23, respectively). Similar findings were noted with the CHA_2_DS_2_-VASc score.

We did not test E:e’ and e’ velocity in a single regression model due to inherent co-linearity between these parameters. However, we compared (using ROC analysis) the diagnostic performance of the predicted probabilities of LAAT derived from an E:e’ based regression model (Model-3) versus an e’ velocity based one (Model-5). This analysis demonstrated that the contribution of E:e’ and e’ velocity to the prediction of LAAT was comparable, as the AUC associated with models 3 and 5 were similar (0.86 and 0.87, respectively; *P* = 0.33).

### E:e’ and e’ velocity association with SEC

One-hundred fourteen subjects (38%) had significant SEC by TEE, including all 19 subjects (100%) with LAAT (Table 2). These patients, as compared to those without SEC, had significantly higher mean E:e’ [14.2 (±5.6) vs. 11.4 (±5.4), *P* = 0.001] and lower e’ velocity [7.7 (±2.5) vs. 9.6 (±3.4) cm/sec, *P* < 0.001]. In multivariate logistic regression analyses, E:e’ and e’ velocity were associated with SEC independent of LVEF, LAVI, CHADS_2_ score, and warfarin therapy (Table 4), with similar results when substituting the CHA_2_DS_2_-VASc for the CHADS_2_ score.

**Table 4:** Multivariate analysis: Predictors of spontaneous echo contrast

		**Odds-Ratio**	**95% CI**	***P *** **value**
**Clinical**	**Model-6**			
	CHADS_2_ Score ( per 1 point)	1.39	1.2 – 1.7	0.001
	Warfarin	2.22	1.4 – 3.7	0.002
**Clinical, E/e’, and 2-D echo**	Model-7*			
	E:e’ (per 1 point)	1.07	1.002 - 1.1	0.04
	LVEF (per 10 point)	0.95	0.8 – 1.2	0.63
	LAVI (per 10 mL/m^2^)	1.53	1.13 – 2.1	0.006
	CHADS_2_ ( per 1 point)	1.22	0.9 – 1.6	0.20
	Warfarin	2.06	1.01 – 4.2	0.048
**Clinical, e’ velocity, and 2-D Echo**	Model-8*			
	e’ velocity (per 1 cm/sec)	0.85	0.7 – 0.97	0.01
	LVEF (per 10 point)	1.0	0.8 – 1.2	0.98
	LAVI (per 10 mL/m^2^)	1.61	1.2 – 2.2	0.002
	CHADS_2_ ( per 1 point)	1.18	0.9 – 1.6	0.29
	Warfarin	1.94	0.94 – 4.0	0.07

**CI:** confidence intervals; **LVEF:** left ventricular ejection fraction (%); **LAVI:** left atrial volume index (mL/m^2^). *The Hosmer and Lemeshow test of the final models (7 and 8) showed good fit (*P* values = 0.25 and 0.75, respectively).

### Diagnostic performance: ROC analyses

**Figure 4:**
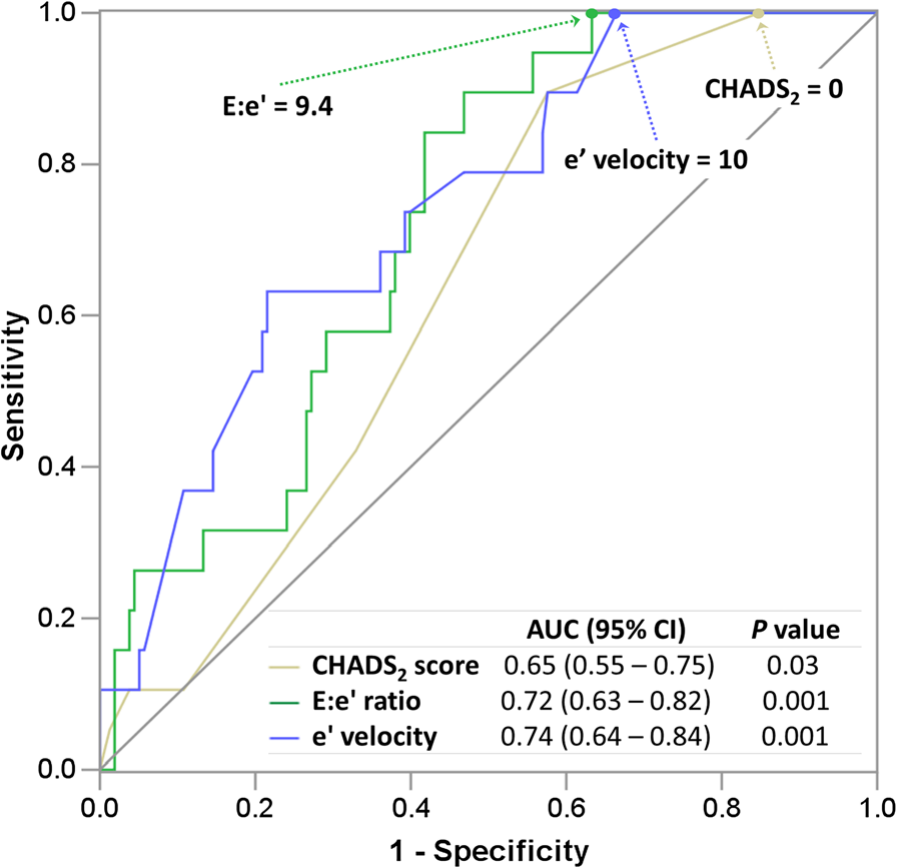
**Receiver operating characteristic curves. AUC**: area under the curve; **CI**: confidence intervals. The receiver operating characteristic curves associated with the E:e’ ratio and CHADS_2_ score were not statistically different (*P* = 0.17); whereas the difference between the curves associated with e’ velocity and the CHADS_2_ score was borderline significant (*P* = 0.07).

The receiver operator characteristics (ROC) curves demonstrated that the E:e’ and e’ velocity have good discriminatory capacity in predicting LAAT with respective areas under the curve (AUC) of 0.72 and 0.74, which trended to be larger than the 0.65 AUC associated with the CHADS_2_ score (*P* = 0.17 and 0.052, respectively), as shown in Figure 4. In this population, the ROC curve point-coordinates identified an E:e’ value of ≥9.4 to have 100% sensitivity and 38% specificity for LAAT; whereas E:e’ ≥ 15 was associated with a specificity of 78% at the expense of a low sensitivity (32%). An e’ velocity ≤10 cm/sec was associated with 100% sensitivity for LAAT (Figure 4). Only a CHADS_2_ score of zero was “protective” from LAAT.

## Discussion

This retrospective cohort study of patients with nonvalvular AF demonstrates that the Doppler diastolic function parameters of E:e’ and e’ velocity are associated with LAAT formation and SEC independent of LVEF, LA volume, and other clinical predictors such as CHADS_2_ (or CHA_2_DS_2_-VASc) score and warfarin use. Furthermore, diastolic function parameters seem to negate the predictive value of the CHADS_2_ or CHA_2_DS_2_-VASc score. If prospectively validated, these findings would indicate that echocardiographic diastolic function parameters may help physicians identify patients at very low risk of LAAT, given their excellent sensitivity. Previous reports have demonstrated that reduced LVEF is associated with LAAT [7,19,20]. Despite the fact that E:e’ and e’ velocity are tightly related to systolic function, [18] we demonstrated that diastolic parameters are associated with LAAT independent of LVEF, likely driven by subjects with impaired diastolic function but preserved LVEF. Furthermore, the study findings are consistent with a recently published study by our group demonstrating that B-type Natriuretic Peptide, a surrogate for LVFP, is predictive of LAAT [21]. These findings are physiologically plausible, as atrial emptying is likely to be diminished with impaired diastolic relaxation and elevated LVFP, leading to atrial blood stasis and thrombus formation. This pathophysiology was the basis for the study hypothesis.

After the inception of this study Iwakura et al. reported that E:e’ is associated with LAAT independent of other echocardiographic parameters such as LVEF and LA dimensions [22]. Our investigation not only confirms these findings, but also demonstrates the independent association between diastolic function parameters and LAAT beyond clinical and other echocardiographic risk predictors.

A previous report has demonstrated that permanent AF is an independent predictor of LAAT, [23] a finding that was not confirmed in our study (Table 1). We suspect that permanent AF status is no more than a confounder for severe left ventricular systolic and diastolic impairment and left atrial enlargement.

This study showed an association between warfarin use and LAAT (Model-1). Clearly, this is not a cause and effect relationship, but rather it is the result of a clinical bias as physicians are likely to use warfarin in patients perceived to be at high risk for LAAT and stroke. This association dissipated once we introduced diastolic parameters, LVEF and LAVI into the regression model.

The authors are cognizant of the small sample size and infrequent LAAT events, which impaired our ability to analyze many covariates within a single regression model. Thus, we meticulously chose the covariates to be analyzed in the logistic regression models and we applied rigorous examination of the goodness of fit of all these models. Nevertheless, it is plausible that some weakly associated parameters may have been missed. Furthermore, the secondary SEC endpoint analyses were consistent with the primary endpoint analyses; thus adding validity to the study conclusions. Moreover, we chose not to test E:e’ and e’ velocity within a single regression model, as the inherent co-linearity between these indices is likely to negate the effects of one another. However, we demonstrated that the predictive values of regression models containing E:e’ and e’ velocity (Models 3 and 5) are similar. Our data does not demonstrate significant superiority of either parameter.

LAVI was shown to be a strong independent predictor of LAAT in multiple reports; [7,8] a finding that was confirmed in this study. However, the predictive value of LAVI was greater when tested with e’ velocity (Model-5) vs. E:e’ (Model-3). This slight discrepancy is explained by the fact that left atrial enlargement is, in part, a manifestation of elevated LVFP assessed by E:e’ [18,24]. Thus, LAVI lost some of its predictive value after adjusting for E:e’. On the other hand, LAVI was a stronger predictor (OR = 1.89 per 10 mL/m^2^) when e’ velocity (rather than E:e’) was included in Model-5 (Table 3). This seems plausible since e’ velocity in itself is not a measure of LVFP. These findings further support the hypothesis that surrogate measures of LVFP such as LAVI, E:e’ or B-type natriuretic peptide are important predictors for LAAT.

Predicting LAAT is certainly important in some clinical scenarios, such as prior to electrical cardioversion [25,26]. Since LAAT is the source of the majority of systemic thromboembolic events in patients with AF, [2,3] it is plausible that diastolic function parameters (E:e’ and e’ velocity) can also help predict embolic stroke. We speculate that, in nonvalvular AF, diastolic impairment and elevated LVFP represent the link between CHADS_2_ risk factors and systemic embolism [27]. We are fully aware that the patient population in this study is distinctly different from that of the AF patient population at large. Furthermore, although LAAT is a precursor of systemic thromboembolism and stroke, [28,29] these endpoints are not necessarily interchangeable. Further validation of this concept in a prospective hard endpoint (stroke) outcome study is warranted.

Our study has a few limitations. First, the retrospective design is an obvious limitation. Second, although the majority of the TTEs were performed within a few days prior to the TEE, some preceded the TEE by as long as 12 months. Thus, the loading conditions at the time of the TTE and TEE may be different for many subjects. However, analysis of the relatively “volume independent” e’ velocity yielded similar results to those observed with E:e’, [30] supporting the study conclusions. Lastly, the small sample size and limited number of events constitute another limitation. Therefore, the findings of this hypothesis-generating study are not applicable clinically at this time, as they demand prospective validation.

## Conclusion

Our investigation demonstrates that the diastolic function indices of E:e’ and e’ velocity are associated with LAAT in patients with nonvalvular AF, independent of clinical and echocardiographic covariates. These findings need to be externally validated, and could potentially be incorporated into a prediction rule that could be utilized clinically for risk stratification for LAAT in patients with nonvalvular AF.

## Abbreviations

AF: Atrial fibrillation; AUC: Area under the curve; CHF: Congestive heart failure; CI: Confidence intervals; E: Mitral inflow early-diastolic flow velocity; e’: Mitral annulus early-diastolic tissue velocity; INR: International normalized ratio; LA: Left atrial; LAAT: Left atrial appendage thrombus; LAVI: Left atrial volume index; LV: Left ventricular; LVEF: Ventricular ejection fraction; LVFP: Left ventricular filling pressure; OR: Odd-ratio; ROC: Receiver operating characteristic; SD: Standard deviation; SEC: Spontaneous echo contrast; TEE: Transesophageal echocardiography; TTE: Transesophageal echocardiogram.

## Competing interests

The authors declare that they have no competing interests.

## Authors’ contributions

RD, MD: Concept, design, statistical analysis, final manuscript approval. EG-S, MD: Echo image interpretation, manuscript drafting and editing. HG, MD: Echo image interpretation, design, manuscript drafting and editing. VN: data collection, cohort definition, data management, manuscript drafting and editing. AD, MD: data collection, data management, manuscript editing. MC, MD: data collection, data management, manuscript editing. NTN, MD: data collection, clinical adjudication. GJK, BS: data collection and management. RT, MD, MBA: critical review and editing. RK, MD, MSc: design, concept, critical review and editing. All authors read and approved the final manuscript.
